# Role of hypoxia inducible factor HIF-1*α* in heart valves

**DOI:** 10.21542/gcsp.2023.9

**Published:** 2023-05-11

**Authors:** Mark Georgy, Kareem Salhiyyah, Magdi H. Yacoub, Adrian H. Chester

**Affiliations:** 1 Magdi Yacoub Institute, Heart Science Centre, Harefield, Middlesex, U.K.; 2 Farah General Hospital, Farah Medical Campus, Mai Ziyadeh Street, Amman, Jordan; 3 National Heart & Lung Institute, Imperial College London, ICTEM Building, Du Cane Road, London

## Abstract

The 2016 Albert Lasker Basic Medical Research Award and subsequently the 2019 Nobel Prize in Physiology or Medicine were awarded to William Kaelin, Jr., Sir Peter Ratcliffe, and Gregg Semenza for their work on how cells sense and adapt to hypoxic conditions. Their work showed that the changes in gene expression, cell metabolism, and tissue remodelling that occur in response to low oxygen concentrations are orchestrated by the transcription factor, hypoxia inducible factor-1*α* (HIF-1*α*). While the effects mediated by HIF-1*α* have been widely studied, its role in heart valves has only recently been investigated. These studies have shown that HIF-1*α* expression is evident in mechanisms that regulate the structure and function of heart valves. These include embryonic development, the regulation of the extracellular matrix, angiogenesis and the initiation of the calcification process. This review provides a background on the role and function of HIF-1*α* in response to hypoxia and a discussion of the available evidence of its involvement in the regulation of heart valves in health and disease.

## Introduction

**Figure 1.**
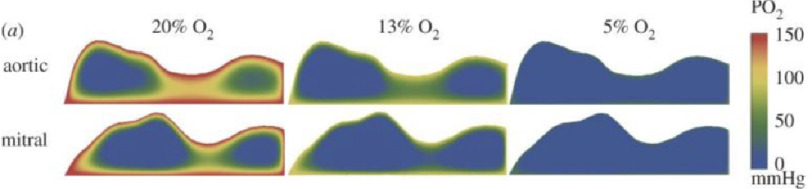
Two-dimensional computational oxygen diffusion maps for aortic and mitral valves. Radial sections of aortic and mitral valves in a relaxed state at different oxygen environments. As external oxygen decreases, the overall leaflet PO_2_ also decreased. Adapted from^45^.

The complex function and durability of heart valves relies upon the function of the endothelial and interstitial cells present in cusp tissue. These cells regulate the integrity of the extracellular matrix and the mechanical properties of the valve via their secretory and contractile properties^1^. As with all metabolically active cells, valve cells require a supply of oxygen in order to function. However, cusp tissue contains very few blood vessels, and those that do exist are located primarily near the annulus of the valve^2^. Computational modelling has shown that diffusion of oxygen from the blood is not sufficient to meet the requirements of the cells in the thickest parts of the cusps^3^. This finding has recently been confirmed by measuring the diffusion of oxygen across porcine aortic valves, taking into account the effect of valve thinning under the distending pressure of the blood during systole^4^. Therefore, cells in the thickest regions of the cusp may function under hypoxic conditions, a situation that will be exacerbated when the valve thickens, as is seen in the early stages of valve stenosis^5^. As oxygen concentrations *in vitro* decrease, PO_2_ levels in aortic and mitral cusp tissue also decrease (Figure 1).

### The need for oxygen regulation

To ensure appropriate oxygen delivery, mammals use complex physiological structural and functional mechanisms to achieve homeostasis^6^. While the rate of oxygen consumption varies between different types of cells and tissues, it is crucial for the cells and organisms to maintain a tight balance of oxygen to meet their requirements. Significant variations occur in the levels of oxygen experienced by different tissues throughout the body, resulting in some cells functioning under hypoxic conditions^7–9^. Humans are exposed to hypoxia when living in high altitudes or while swimming, diving, or exercising^10,11^. Physiologically, embryonic development occurs under hypoxic conditions and stem cells are maintained in hypoxic niches to protect them from oxidative damage^12^. Pathologically, hypoxia is associated with conditions such as ischaemia, wound healing, cancer, and many others.

Since hypoxia is not an unusual event in the cellular life cycle, cells have intrinsic pathways to tolerate, cope, adapt, and reverse hypoxia. Cellular responses to hypoxia usually start by altering oxygen consumption and reducing the metabolic rate^10^, followed by increased glycolysis and glucose transportation^13^. Cell survival pathways and growth factors like insulin-like growth factor-2 (IGF2) and transforming growth factor-*α* (TGF-*α*) are then activated to help cells tolerate the hypoxic insult^14,15^.

The majority of oxygen homeostasis is mediated through the hypoxia-inducible factor-1*α* (HIF-1*α*). HIF-1*α*, discovered by Prof Greg Semenza and Guang Wang from Johns Hopkins in 1992^16,17^, is a factor that is induced by hypoxia and induces a several hundred-fold increase in transcriptional expression of EPO mRNA through binding to 3’ enhancer sequence, later defined as hypoxia response element (HRE)^18^. It is considered the master oxygen regulator, and the most important player in oxygen homeostasis^19^.

### Activation of HIF-1*α* in response to hypoxia

HIF-1*α* is a constitutively expressed transcription factor that, under normoxic conditions degrades rapidly with a half-life of about 1 min^20^. HIF-1*α* is hydroxylated by prolyl hydroxylase domain protein (PHD) using oxygen as a substrate and is then rapidly degraded. Under hypoxic conditions, PHD is unable to hydroxylate the prolene residues on HIF-1*α* and consequently, binding to the product of von Hippel Lindau (pVHL), allowing HIF-1*α* to stabilise in the cytoplasm.

**Figure 2.**
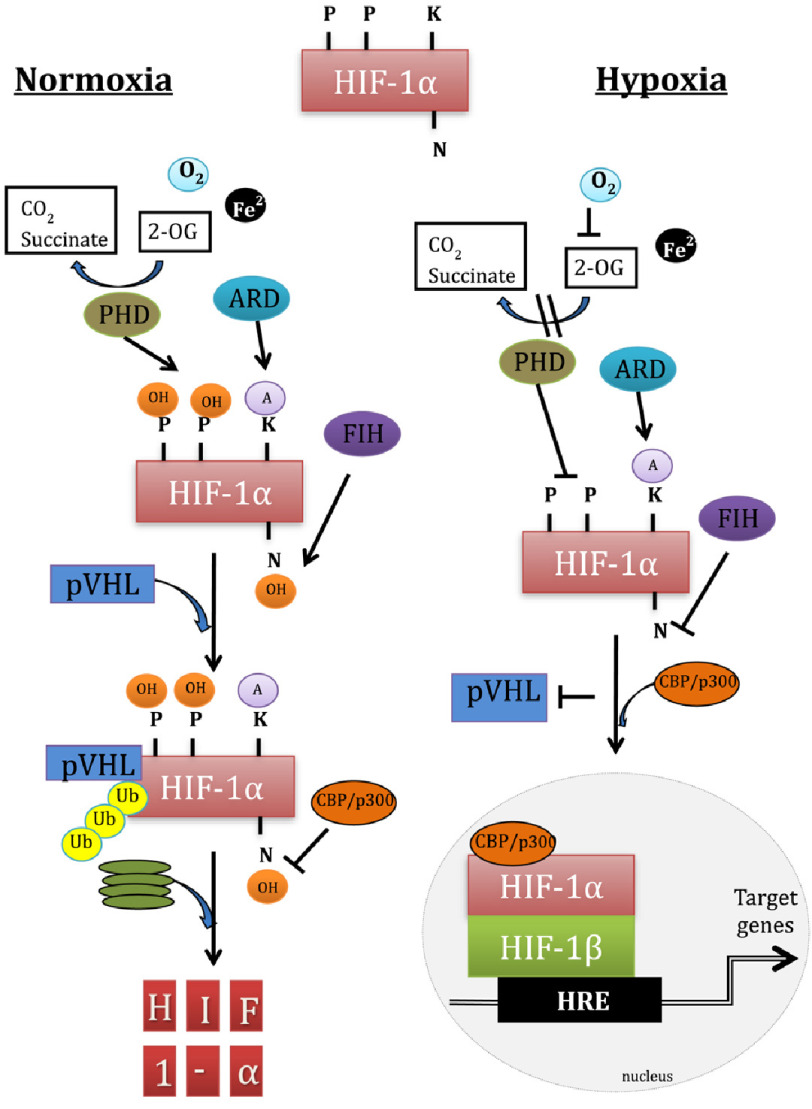
Changes to HIF-1*α* under both hypoxic and normoxic conditions. Under normoxia, 2 proline (P) residues 402 and 564 of HIF-1*α* are hydroxylated (OH) by prolyl hydroxylase domain (PHD) as it transforms 2-oxoglutarate (2-OG) to CO_2_ and succinate in an O_2_ and Fe ^2+^ dependent manner. Factor-inhibiting hypoxia-inducible factor (FIH) will hydroxylate asparagine 803 (N). In addition, lysine 532 (K) will be acylated (A) by ARD which partially dependent on O_2_. Hydroxylated HIF-1*α* binds to pVHL and forms a complex with E3 ubiquitin (Ub) ligase, leading to proteasomal degradation. Under hypoxia, lack of O_2_ will inhibit the function of PHD and FIH. Un-hydroxylated HIF-1*α* is stabilised by virtue of its inability to bind pVHL. Stable HIF-1*α* translocates to the nucleus and binds to HIF-1*β* forming a dimer. Coactivator CBP/p300 will bind to the dimer through nonhydroxylated (N). The HIF dimer will attach to hypoxia response elements (HRE) and induce transcription of target genes. Adapted from^59^.

Stable HIF-1*α* in the cytoplasm will translocate to the nucleus. There, it will form a dimer with a closely related subunit, HIF-1*β*, otherwise known as the aryl hydrocarbon nuclear translocator (ARNT). The active dimer of HIF-1*α* and HIF-1*β* will then recruit various transcriptional co-activators. This will allow the HIF complex to bind to specific HRE sequences in the enhancer or promoter regions of various target genes and activate their transcription (Figure 2).

Over 1000 genes have been shown to be modulated by the HIF-1*α* pathway. Some of the well-known genes include VEGF^21^, platelet derived growth factor^22^, glucose transporter-1^23^, and erythropoietin^16^. As a result, HIF-1*α* has been implicated in the pathophysiology of a number of different diseases including cancer, pulmonary hypertension, inflammatory bowel disease, ischaemic stroke and diabetes^24–30^.

With respect to the heart, analysis of animal models suggests that via activation of homeostatic mechanisms that regulate oxygen delivery and utilization, HIF-1*α* plays a critical protective role in the pathophysiology of ischemic heart disease and pressure-overload heart failure. Tissue hypoxia or ischemia resulting from coronary stenosis induces HIF-1*α* activity, which is required to produce angiogenic growth factors that stimulate vascular remodelling to increase blood flow through collateral vessels. This subject has recently been extensively reviewed^19,31,32^. This article will specifically focus on the role of HIF-1*α* in the valves of the heart.

### Expression of HIF-1*α* in heart valves

**Figure 3.**
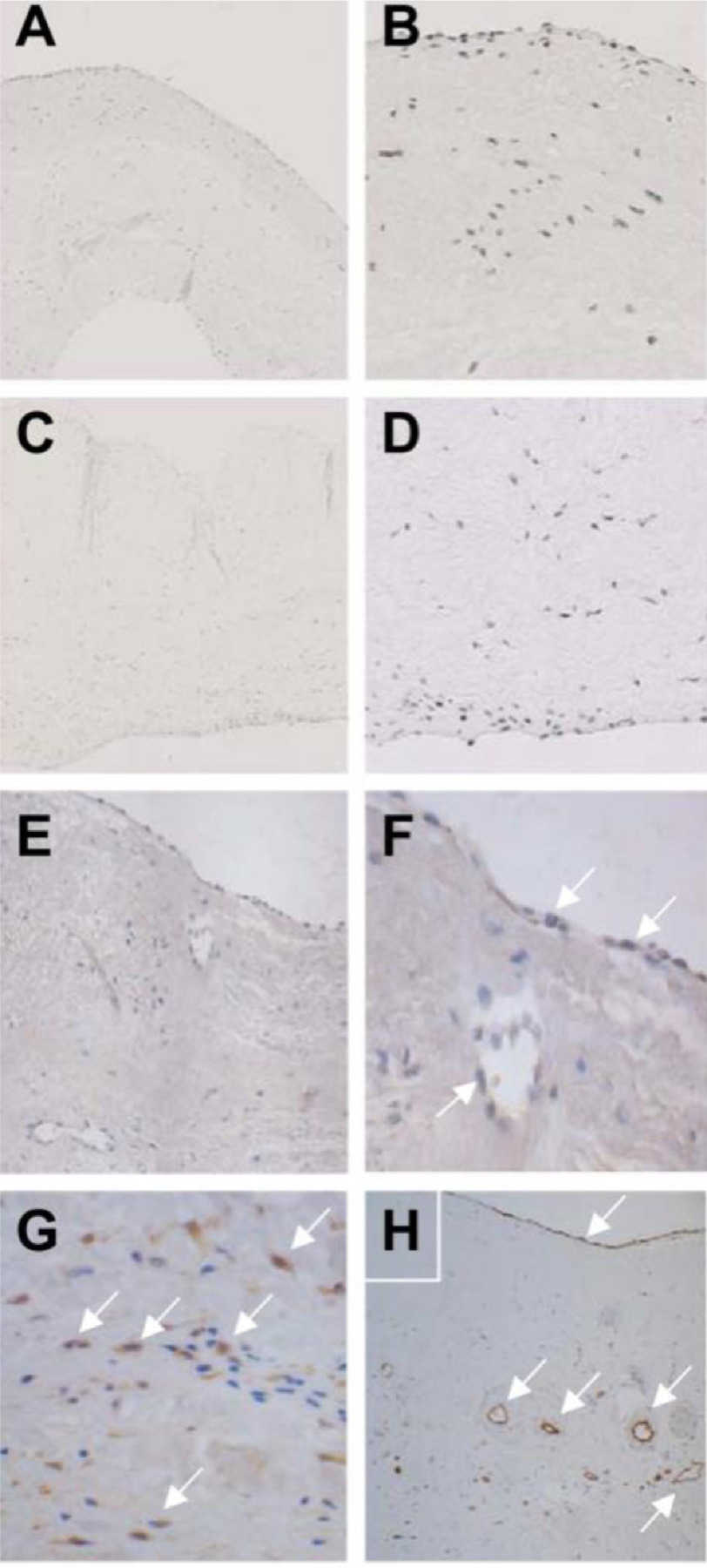
Expression of HIF-1*α* in sections of mitral valves. *A* and *B* Normal mitral valves; *C* and *D* myxomatous mitral valves; E, F, G and H rheumatic mitral valves. Normal and myxomatous valves showed very weak staining for HIF-1*α*. In sections from rheumatic valves staining for HIF-1*α* was present on surface of the valves and on cells lining vessels within rheumatic mitral valves. Staining was also present on interstitial cells. Modified from^34^.

**Figure 4.**
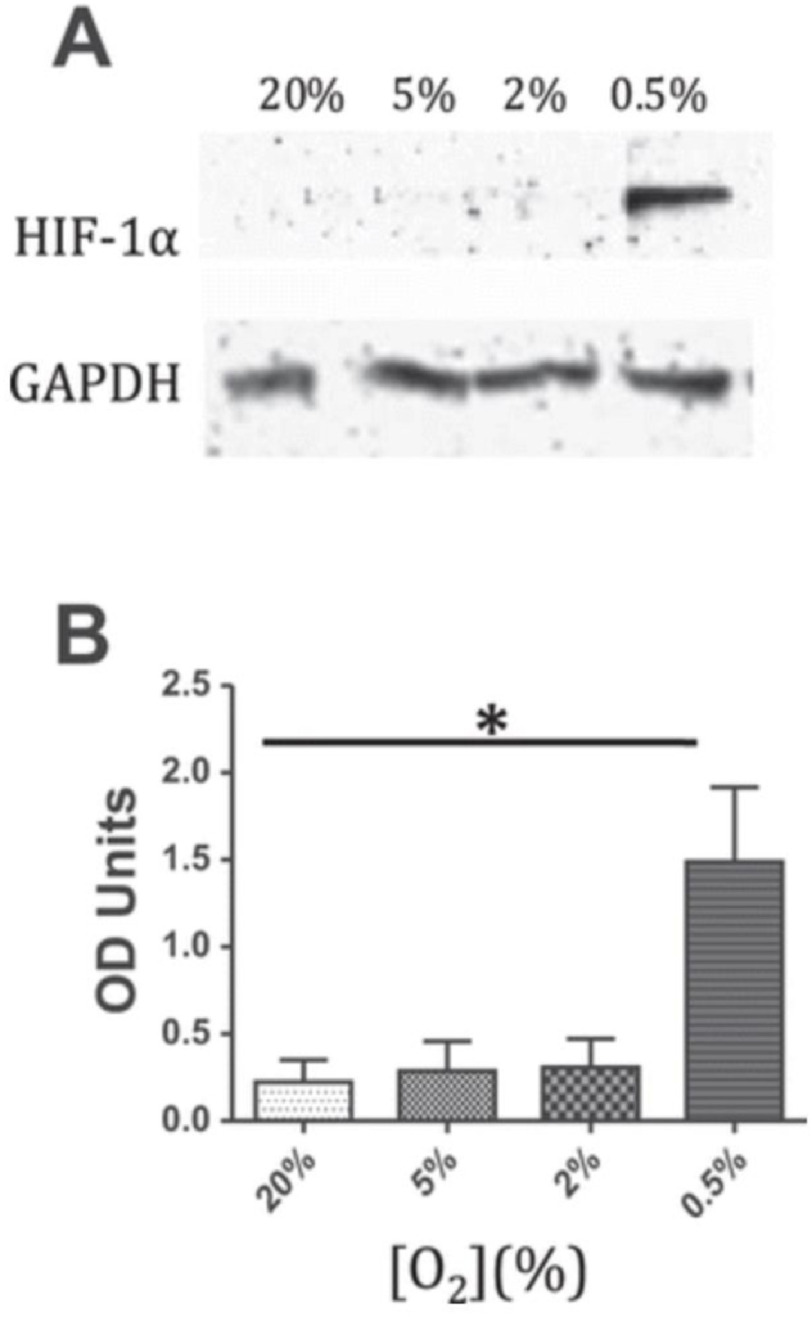
Expression of HIF-1*α* protein in response to hypoxia. Mitral valve interstitial cells were exposed to different concentrations O_2_ for 72 h. A. Western blots and B. quantification of band intensity (*P < 0.05.) showed expression of HIF-1*α*. Modified from^34^.

The expression of HIF-1*α* has been reported in degenerative human aortic valves. Calcified regions of valves showed upregulation of HIF-1*α* and VEGF, which were co-localised with each other in thickened regions of the valve. It was also shown that areas adjacent to calcific lesions are associated with the morphological features of a neovasculature^33^. HIF-1*α* expression has also been reported in rheumatic mitral valves, where it was seen at greater levels of expression than that seen in normal or myxomatous degenerated valves (Figure 3)^34^. Experientially HIF-1*α* can be induced in valve interstitial cells by progressively lowering the oxygen concentration (Figure 4). Exposure of aortic and mitral valve cusp to tissue hypoxia led to a loss of layer stratification and elevated levels of HIF-1*α* expression.

#### Valve development

HIF-1*α* is understood to play a key role in multiple facets of cardiac development. HIF-1*α* is expressed in migrating neural crest cells and during ventricular formation. Its lack of expression is associated with cardiac abnormalities, such as the failure of the bilateral myocardial cells to coalesce into a single central heart tube, resulting in the presence of two independent hearts. namely cardia bifida^35^. It has been recorded that HIF-1*α* was localized in chick aortic valves during the late embryonic period at locations where increased levels of glycosaminoglycan were identified.

Additionally, prenatal hypoxic conditions, which result in increased levels of HIF-1*α*, were identified to be imperative for the nuclear localization of the Sox9 transcription factor, thus providing a foundational understanding in the development of heart valves being linked to chondrogenic mechanisms^36^. HIF-1*α* signalling via the expression of VEGF is involved in the developing cardiac outflow tract, and inhibition of this pathway causes defects in the formation of the outflow tract that resemble congenital conotruncal heart defects such as truncus arteriosus, tetralogy of Fallot, interrupted aortic arch, transposition of the great arteries, and double outlet right ventricle^37^.

Embryonic valve development, post-natal valve homeostasis and, in some instances, structural valve dysfunction can all be mediated by endothelial-to-mesenchymal transition^38^. In coronary artery endothelial cells, hypoxia induced expression of HIF-1*α* induces the expression of SNAIL, a regulatory transcription factor for endothelial-to-mesenchymal transition. Whether this mechanism occurs in valve endothelial cells remains to be determined. Postnatally, upon achieving normoxia, murine valves showed that expression of HIF-1*α* persisted on valve endothelial cells, which was associated with remodelling of the extracellular matrix and arrest of valve interstitial cells proliferation^36^.

#### Regulation of the ECM

While heart aortic valve cells can respond to a wide range of mechanical stimuli to produce matrix proteins^39,40^, it has also been shown that hypoxia has the capacity to be a stimulus for matrix protein secretion. In a variety of different cell types hypoxia can induce the upregulation of ECM homeostatic related genes^41^. However, exposing mitral valve interstitial cells to hypoxia induced a reduction in the expression of collagen and sulphated glycosaminoglycans, an effect that was associated with the expression of HIF-1*α*^34^. Porcine aortic valve interstitial cells from aged pigs exposed to hypoxia demonstrated raised expression of HIF-1*α* and the complex of matrix metalloprotease 9 (MMP9) and neutrophil gelatinase-associated lipocalin (NGAL)^42^.

The complex of NGAL with MMP9 serves to stabilise the metalloprotease, resulting in greater gelatinolytic activity of MMP-9 on the extracellular matrix^43^. Taken together, these studies suggest that in valve interstitial cells there is a link between HIF-1*α* and the regulation of the valve extracellular matrix. These mechanisms may become relevant as the valve cusps thicken in the early stages of the disease process and regions of the valve cusps become hypoxic.

#### Angiogenesis

HIF-1*α* has been shown to directly modulate a plethora of transcription factors including the pro-angiogenic transcription factors such as VEGF^21^. In this context, a role for HIF-1*α* has been established in tumour angiogenesis, new vessel growth during cardiac and cerebral ischemia^19,44^. In cases of valvular stenosis, aortic valve thickening leads to hypoxic conditions within the thickest parts of the valve cusp, potentially leading to increased HIF-1*α* expression, subsequent increased VEGF expression, and thus neovascularization^33^. When comparing atmospheric (20% O_2_) with physiological levels of oxygen (13% O_2_), exposure of aortic and mitral valves to the physiological levels of oxygen showed significantly raised levels of the angiogenic transcription factor VEGFR2+ along with HIF-1*α*^45^.

**Figure 5.**
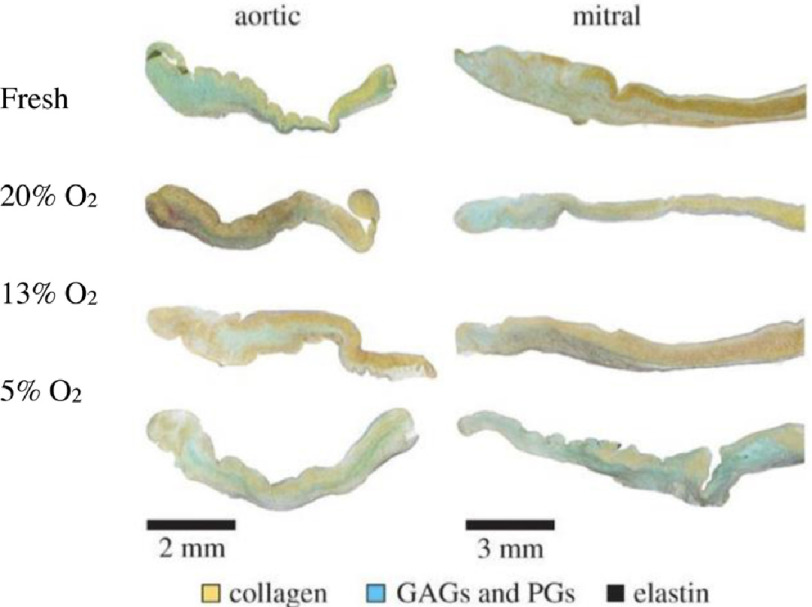
Histological assessment of effect of oxygen concentration on the extracellular matrix. Expression of collagen, glycosaminoglycans (GAGs) proteoglycans (PG) and elastin in aortic and mitral valve leaflets that were exposed to a lower oxygen environment. Collagen content was reduced at low oxygen concentrations when compared with the 20% oxygen group. Adapted from^45^.

In the thicker mitral valve cusps, there was evidence of a concomitant expression of angiogenic factors and increased cell death, that was not apparent in the thinner aortic valve cusps, suggesting that the regulation of angiogenesis may differ between the two types of valve (Figure 5). The increased HIF-1*α*, VEGF and regions of neovascularisation expression are associated with regions of calcification in stenotic aortic valves.

#### Calcification

The cellular components of the aortic valve are known to participate in the processes that lead to aortic sclerosis and eventually to the development of aortic stenosis. Medical strategies to treat the disease with pharmacological intervention are limited. In order to develop new pharmacological treatments, there is a need to understand causative factors and/or mediators that are involved in the onset and progression of this condition. In this respect HIF-1*α* constitutes potentially novel therapeutic target.

The closely related molecule HIF-2*α*, which is an oxygen-sensitive transcription factor that has different physiological roles to HIF-1*α*^46^, is expressed in cells recruited to the sites of calcification and stimulates Sox9 and Runx2, two molecules implicated in the pathogenesis of calcific valve disease^47–49^. These effects were supported by the observation that the cardiac glycoside digoxin, that inhibits the expression of HIF-2*α*, could decrease the amount of calcification in soft tissues^49^. This study illustrates that the proinflammatory microenvironment, rather than hypoxia, can act as physiological stimulus for expression of HIF molecules. Indeed, studies with human aortic valve interstitial cells have shown the ability of interferon-gamma (IFN-g) to induce the expression of HIF-1*α* under atmospheric oxygen conditions, an effect potentiated by the Toll-like receptor-4 agonist lipopolysaccharide (LPS)^50^. In this study IFN-g and LPS were able to induce valve interstitial cells to adopt an osteogenic phenotype. Further studies by these investigators showed that Tumour Necrosis Factor-a (TNF-a), but not IFN-g, could also induce HIF-1*α* expression in cultured aortic valve endothelial cells. IFN-g triggered cytokine secretion and adhesion molecule expression in valve endothelial and interstitial cells^51^.

The different patterns of blood flow experienced either side of the aortic valve have also been implicated in expression of HIF-1*α* . It has been shown that disturbed patterns of flow, characteristic of the aortic side of the valve, are associated with expression of HIF-1*α*, miR-483, ubiquitin E2 ligase C and von Hippel-Lindau protein, which have all been shown to be flow-sensitive molecules^52^. Under conditions of disturbed flow, von Hippel-Lindau Protein is degraded by ubiquitin E2 Ligase C, causing HIF-1*α* levels to rise. In addition, levels of miR-483 are reduced by disturbed flow, which results in an increase in ubiquitin E2 Ligase C activity and a rise in HIF-1*α* expression. Moreover, treatment with the HIF-1*α* inhibitor (PX478) significantly reduced porcine aortic valve calcification in static and disturbed flow conditions^53^.

In a similar manner, a role for HIF-1*α* is also implicated in other calcific diseases in the vasculature^54,55^. Osteogenic transdifferentiation of vascular smooth muscle cells is promoted by raised levels of inorganic phosphate via the activation HIF-1*α* under normoxic conditions^56^. This effect of inorganic phosphate is accelerated by Daprodustat, a prolyl hydroxylase inhibitor, that increases erythropoiesis via activation of HIF-1*α*^57^. HIF-1*α* has been shown to be expressed in atherosclerotic lesions and femoral endarterectomy specimens^58^. Expression was seen in macrophages, macrophage-derived foam cells and some smooth muscle cells.

## Conclusion

Whether the stimulus is reduced oxygen concentration, cytokines or disturbed patterns of flow, HIF-1*α* has the capacity to induce physiological and pathophysiological changes in heart valves. Consideration needs to be given to the fact that valves on the left and right side of the heart reside in extremes of oxygen concentration (13.3% oxygen for aortic and mitral valves; 0.5% oxygen for pulmonary and tricuspid valves, and that most *in vitro* studies are carried out at atmospheric conditions (21% oxygen). Given that valve cusp thickening is recognised as an early event in the disease process^5^, this will gradually impair the diffusion of oxygen to the thicker regions of the valve cusp and potentially lead to the expression of HIF-1*α*. Whether this is the trigger for the expression of angiogenic factors and the induction of valve interstitial cell differentiation and ultimately calcification warrants further investigation.

Elucidating how and when HIF-1*α* is expressed under physiological and pathophysiological conditions, and determination of the functional and structural changes it induces in heart valves will lead to the identification of new pharmacological targets which may lead to the development of new treatment strategies for heart valve disease.
